# The diagnostic accuracy of multiparametric MRI for detection and localization of prostate cancer depends on the affected region

**DOI:** 10.1002/bco2.62

**Published:** 2020-11-28

**Authors:** Martina Martins, Stefano Regusci, Stephane Rohner, Ildiko Szalay‐Quinodoz, Georges‐Antoine De Boccard, Louise Strom, Gerjon Hannink, Sonia Ramos‐Pascual, Charles Henry Rochat

**Affiliations:** ^1^ Swiss International Prostate Center Geneva Switzerland; ^2^ ImageRive, Institut de Radiologie Spécialisée Geneva Switzerland; ^3^ Clinique Générale Beaulieu Geneva Switzerland; ^4^ ReSurg SA Nyon Switzerland; ^5^ Radboud University Medical Center Nijmegen Netherlands

**Keywords:** diagnostic accuracy, localization, magnetic resonance imaging, mpMRI, prostate cancer, PI‐RADS, radical prostatectomy

## Abstract

**Objectives:**

To determine the diagnostic accuracy of 3T multiparametric magnetic resonance imaging (mpMRI) for detecting and locating prostate cancer (PCa) on Dickinson's 27‐sector map, using histopathology specimens from radical prostatectomy (RP) as the reference standard.

**Patients and methods:**

The authors studied a continuous series of 140 patients who underwent RP over three consecutive years. Prior to RP, all patients had mpMRI for detection and localization of PCa and further assessment by biopsy. To minimize the potential of disease progression, 25 patients were excluded because the interval between mpMRI and RP exceeded 6 months, which left 115 patients eligible for analysis. The mpMRI findings were reported using the Prostate Imaging‐Reporting and Data System (PI‐RADS) v2, considering PI‐RADS ≥ 3 to indicate PCa. The histopathology findings from RP specimens were graded using the Gleason scoring system, considering Gleason ≥ 6 to indicate PCa. The location of the tumors was mapped on Dickinson's 27‐sector map for both mpMRI and histopathology and compared by rigid sector‐by‐sector matching.

**Results:**

The cohort of 115 patients eligible for analysis was aged 66.5 ± 6.0 years at RP. Of the 3105 sectors analyzed, there were 412 true positives (13%), 28 false positives (1%), 68 false negatives (2%), and 2597 true negatives (84%). Across the 27 sectors of the prostate, mpMRI sensitivity ranged from 50% to 100% and specificity from 96% to 100%, while PPV ranged from 50% to 100%, and NPV from 91% to 100%. For the anterior prostate, mpMRI had a sensitivity of 80% (CI, 71%‐86%), specificity of 99% (CI, 99%‐100%), PPV of 91% (CI, 83%‐95%), and NPV of 99% (CI, 98%‐99%). For the posterior prostate, mpMRI had a sensitivity of 88% (CI, 84%‐91%), specificity of 98% (CI, 97%‐99%), PPV of 94% (CI, 92%‐96%), and NPV of 96% (CI, 94%‐97%). Overall, mpMRI had a sensitivity of 86%, specificity of 99%, PPV of 94%, and NPV of 97%.

**Conclusions:**

The accuracy of mpMRI in detecting and locating prostate tumors depends on the affected region, but its high NPV across all sectors suggests that negative findings may not need corroboration by other techniques.

## 1 INTRODUCTION

Prostate cancer (PCa) is the most frequently diagnosed cancer in men, accounting for 20% of cancer diagnoses, and is the second most common cause of cancer‐related death in this population.^1^ Clinically significant PCa (csPCa) does not have a universally agreed definition, although it is most commonly defined histopathologically using the criteria established by either Wolters et al.^2^ or the Prostate Imaging‐Reporting and Data System (PI‐RADS).^3^ A positive finding on digital rectal examination (DRE) and/or a prostate‐specific antigen (PSA) result of ≥ 4 ng/mL raises the suspicion of csPCa.^4^ Multi‐parametric magnetic resonance imaging (mpMRI) is increasingly used for noninvasive detection of PCa, as well as its staging and localization.^5^ With improved diagnostic accuracy, mpMRI can guide and enhance biopsy planning,^6^ as well as inform appropriate treatment options, such as focal ablative therapies^7^ and nerve‐sparing surgery.^8^

To standardize mpMRI reporting, PI‐RADS was proposed in 2012, introducing a scoring system to identify and locate prostate tumors, as well as predict the likelihood of csPCa. The recommended sector map, proposed by Dickinson et al.,^9^ divided the prostate into 27 sectors to facilitate assessment in predefined regions. PI‐RADS v2 was published in 2015, introducing the concept of “dominant sequences” to simplify mpMRI evaluation, and updated the 27‐sector map to a 39‐sector map, separating the central zone of the prostate.^10^

The diagnostic accuracy of mpMRI has been investigated against histopathological specimens obtained by transperineal or transrectal ultrasound‐guided (TRUS) biopsy,^11^, ^12^, ^13^ template mapping prostate biopsy (TMPB)^14^, ^15^ and/or radical prostatectomy (RP).^16^, ^17^, ^18^, ^19^, ^20^ While each technique for histopathological sampling has its own limitations,^21^ RP is often considered the “gold standard,” as it provides a definitive evaluation of the prostate gland.^17^

Some studies investigating the accuracy of mpMRI against RP specimens described tumor location using prostate anatomical zones (peripheral or transitional),^19^ anatomical “levels” (base, mid‐gland and apex)^15^ or PI‐RADS sector maps.^22^ To the authors’ knowledge, however, no contemporary study reported the accuracy of 3T mpMRI with regards to the exact localization of tumors stratified using Dickinson's 27‐sector map. This study, therefore, aimed to determine the diagnostic accuracy of 3T mpMRI for detecting and locating PCa (PI‐RADS ≥ 3) on Dickinson's 27‐sector map, using histopathology specimens from RP as the reference standard.

## 2 PATIENTS AND METHODS

### 2.1 Patient selection

The authors retrospectively analyzed the records of 140 consecutive patients who underwent RP under the care of the senior surgeon (CHR) between March 2015 and May 2018. Prior to RP, all patients had an initial suspicion of PCa, indicated by PSA ≥ 4 ng/mL and/or positive DRE, followed by detection and localization using mpMRI and further assessment by TRUS or transperineal biopsy, using both a targeted and randomized sampling approach. All mpMRIs, biopsies and RP procedures were performed at the same institution. None of the patients received any treatment for PCa between mpMRI and RP procedures. Twenty‐five patients were excluded from the study because the interval between mpMRI and RP exceeded 6 months, to minimize the potential of significant disease progression following mpMRI, leaving 115 patients eligible for analysis. All patients provided written informed consent for the use of their data and images for research and publication purposes, and the study was approved by the institutional review board.

### 2.2 MRI technique

All prostate mpMRIs were acquired with the patient in the supine position (feet first), using a 3T unit (Achieva, Philips Healthcare, Eindhoven, NL) with an external pelvic phased‐array coil (TorsoXL coil, Philips Healthcare, Eindhoven, NL) but without an endorectal coil. An antispasmodic agent (2 mls of 20 mg/mL hyoscine butylbromide; Buscopan®, Boeringer) was administered intravenously, to minimize peristalsis of the bowel and thereby reduce movement artifact on the image. The imaging protocol used was in accordance with the PI‐RADS v2 guidelines, with intravenous contrast injection of 0.1 ml/kg gabobenate dimeglumine (MultiHance®, Bracco Eisai, Tokyo, Japan), administered through a peripheral vein at a rate of 4 mL/s. The sequences acquired before contrast injection included axial, sagittal and coronal T2‐weighted fast spin‐echo (FSE), an axial T1‐weighted FSE, axial diffusion‐weighted images (DWI) using b0, b100 and b1500 to generate the apparent diffusion coefficient (ADC) map, and a separate high b value DWI (b2000s/mm^2^). During contrast injection, an axial three‐dimensional (3D) FSE dynamic contrast enhanced (DCE) sequence was acquired. After contrast injection, an axial T1‐weighted FSE sequence was acquired.

### 2.3 Imaging analysis

Each tumor within the prostate gland was identified and evaluated by the same radiologist with 15 years’ experience in prostate MRI and graded as per PI‐RADS v2 to report likelihood of csPCa (1: highly unlikely, 2: unlikely, 3: equivocal, 4: likely, and 5: highly likely). Therefore, the present study considered PI‐RADS ≥ 3 in a given sector to indicate PCa in that sector, and PI‐RADS ≥ 4 in a given sector to indicate csPCa in that sector. Each tumor was then mapped onto Dickinson's 27‐sector map, thereby assigning a PI‐RADS grade to each sector. Tumor‐nodes‐metastasis (TNM) staging criteria were used to report presence of extra prostatic extension (EPE), according to the American Joint Committee on Cancer (AJCC),^23^ with pT3 staging considered positive for EPE.

### 2.4 Radical prostatectomy histopathology analysis

All patients underwent robotic‐assisted laparoscopic RP by two urological surgeons. Histopathologic whole‐mount specimens were prepared following the College of American Pathologists “Protocol for the Examination of Radical Prostatectomy Specimens From Patients With Carcinoma of the Prostate Gland”.^24^ Specimens were then assessed for presence of tumor and EPE by four experienced genitourinary pathologists, who were blinded to the results of mpMRI. Individual tumors were graded using the Gleason scoring system^25^ and their location was outlined onto Dickinson's 27‐sector map, thereby allocating a Gleason score to each sector. Gleason grade groups (GG) were defined as follows: Gleason 3 + 3 = GG1, Gleason 3 + 4 = GG2, Gleason 4 + 3 = GG3, Gleason 4 + 4 = GG4, and Gleason > 4+4 = GG5. csPCa is defined by Wolters et al.^2^ as (i) Gleason 7‐10 with > 5% grade 4 and ≥ 0.7 cc; (ii) Gleason 6 and ≥ 1.3 cc; (iii) pT stage 3a or greater (EPE); and (iv) nodal metastasis. The present study considered Gleason score ≥ 6 in a given sector to indicate PCa in that sector, and Gleason score ≥ 7 in a given sector to indicate csPCa in that sector.

### 2.5 Correlation between mpMRI and histopathology

**FIGURE 1:**
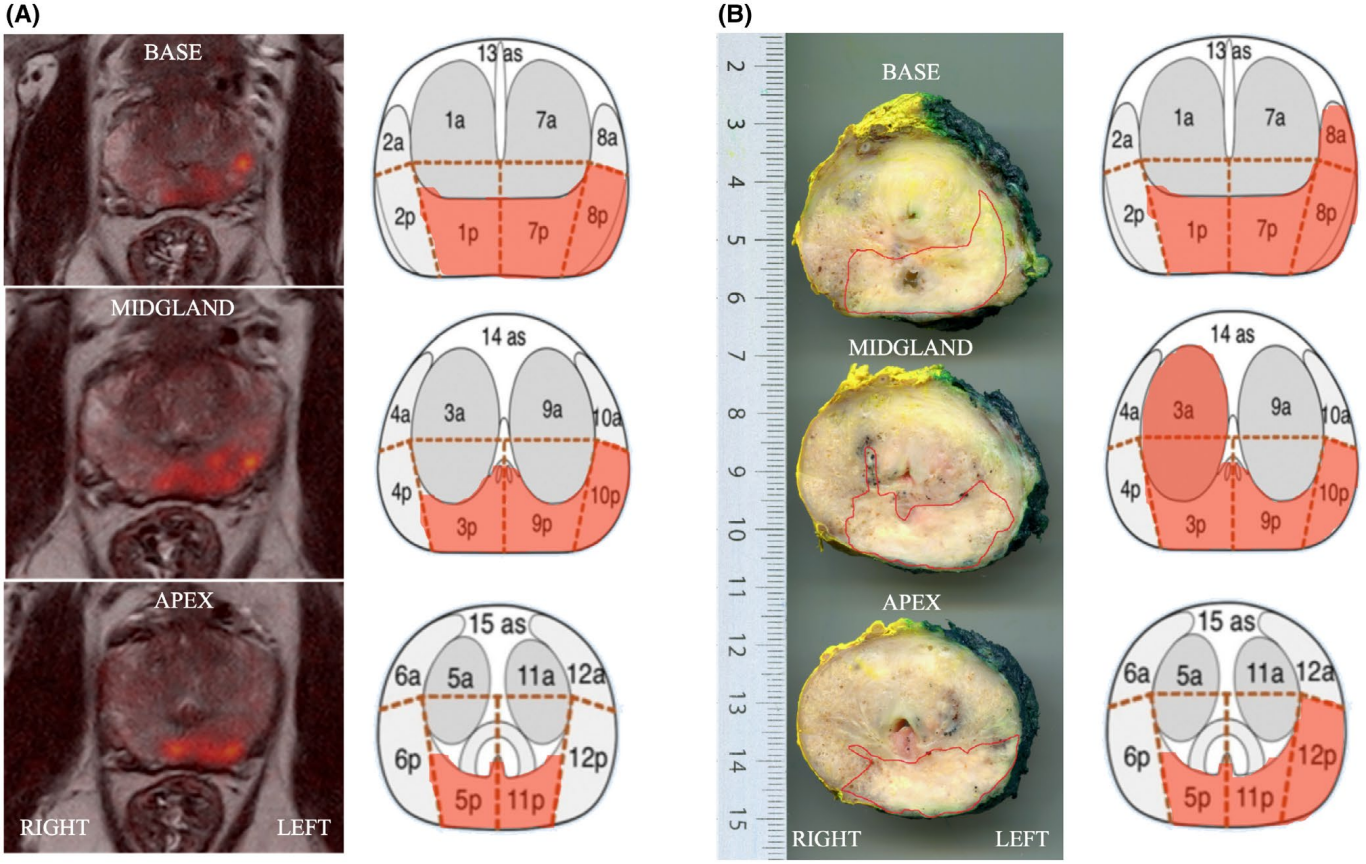
(A) Axial T2‐weighted fast spin‐echo fused with DWI b2000s/mm (using Osirix software), demonstrating a PI‐RADS 5 score in the left posterolateral base, midgland, and apex, which is illustrated in Dickinson's 27‐sector map. (B) Histopathologic whole‐mount specimen from radical prostatectomy demonstrating a Gleason score 3 + 4 tumor, in the left posterolateral base, midgland, and apex, which is illustrated in Dickinson's 27‐sector map

Histopathology findings were used as the reference standard for tumor detection and the findings on mpMRI and histopathology were compared by rigid sector‐by‐sector matching^18^ (Figure 1). True positives (TP) indicate PCa observed on both mpMRI and histopathology, false positives (FP) indicate PCa observed on mpMRI but not histopathology, false negatives (FN) indicate PCa observed on histopathology but not mpMRI, and true negatives (TN) indicate absence of PCa on both mpMRI and histopathology.

### 2.6 Statistical analysis

The accuracy of mpMRI at detecting PCa (PI‐RADS ≥ 3 and Gleason ≥ 6) and csPCa (PI‐RADS ≥ 4 and Gleason ≥ 7) in each sector was expressed in terms of sensitivity/specificity and positive predictive value (PPV)/negative predictive value (NPV), with 95% confidence intervals (CI). All values were calculated for the 27 sectors, as well as for the anterior prostate, the posterior prostate, and the prostate overall, by summing the numbers of respective TPs, FPs, FNs, and TNs. The accuracy of mpMRI at detecting EPE was also expressed in terms of sensitivity/specificity and PPV/NPV for the entire prostate. Statistical analyses were performed using R version 3.6.2 (R Foundation for Statistical Computing, Vienna, Austria).

## 3 RESULTS

The cohort of 115 patients eligible for analysis had a mean (± standard deviation) age of 66.5 ± 6.0 years (range, 50.9‐77.8) at the time of RP. The time interval from mpMRI to RP was 101 ± 36 days (range, 18‐177). Index tumor volume measured on mpMRI was 4.9 ± 4.7cc (range, 0.4‐22.5) and volume of the prostate gland measured by histopathology was 45.8 ± 15.9cc (10.0‐104.0). Histopathology revealed csPCa in 114 patients, and only 1 patient who had Gleason score 3 + 3, tumor volume <0.7cc, and EPE negative (Table 1). According to histopathology, the Gleason score was 3 + 3 in 43 sectors (9.0%), 3 + 4 in 282 (58.8%), 4 + 3 in 121 (25.2%), 4 + 4 in 2 (0.4%), and 4 + 5 in 32 (6.7%). According to mpMRI, the PI‐RADS score was 3 in 70 sectors (15.9%), 4 in 118 (26.8%), and 5 in 252 (57.3%).

**TABLE 1:** Patient (n = 115) demographics

	Mean ± SD	Median	Range
	n (%)		
Age at RP (years)	66.5 ± 6.0	67.1	(50.9‐77.8)
Index tumor volume (cc)	4.9 ± 4.7	3.6	(0.4‐22.5)
Time from mpMRI to RP (days)	101 ± 36.1	98.0	(18‐177)
Mean prostate volume (cc)	45.7 ± 15.9	45.0	(10.0‐104.0)
Clinically significant cancer^a^	114 (99.1%)		

Abbreviations: cc, cubic centimeter; mpMRI, multiparametric magnetic resonance imaging; PI‐RADS, Prostate Imaging‐Reporting and Data System; RP, radical prostatectomy; SD, standard deviation.

^a^ Clinically significant cancer was based on Wolters criteria: (i) Gleason 7‐10 with > 5% grade 4 and ≥ 0.7 cm^3^; (ii) Gleason 6 and ≥ 1.3 cc; (iii) pT stage 3a or greater; and (iv) nodal metastasis.

### 3.1 Detection of PCa

For the detection of PCa (PI‐RADS ≥ 3 and Gleason ≥ 6), of the 3105 sectors analyzed (115 × 27 sectors), histopathology from RP specimens reported 480 positive sectors (15.5%), while mpMRI reported 440 positive sectors (14.2%). There were 412 TPs (13%), 28 FPs (1%), 68 FNs (2%), and 2597 TNs (84%) (Table 2). The most frequently positive sectors were 10p (n = 57), 4p (n = 42), and 9p (n = 41), and the least frequently positive sectors were 7a (n = 3), and 1a, 13as, 9a, and 11a (n = 4). In the 68 sectors where PCa was not observed on mpMRI (FNs), the Gleason score was never more than 7 (3 + 3 in 17, 3 + 4 in 43, and 4 + 3 in 8).

**TABLE 2:**
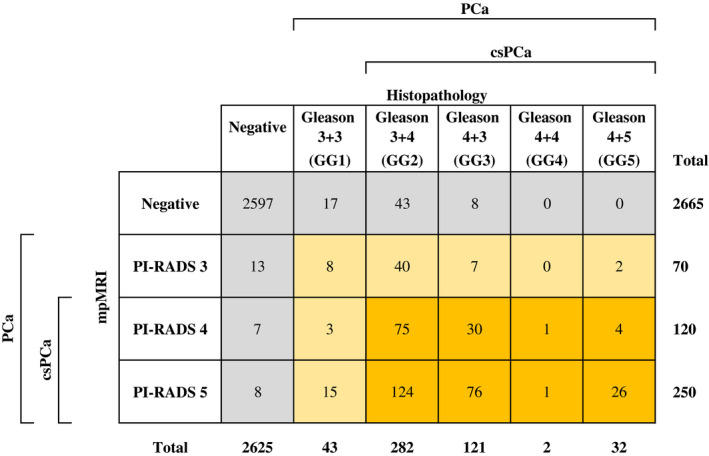
mpMRI correlation with histopathology

Abbreviations: GG, group grade; mpMRI, multi‐parametric magnetic resonance imaging; PI‐RADS, Prostate Imaging‐Reporting and Data System.

**FIGURE 2:**
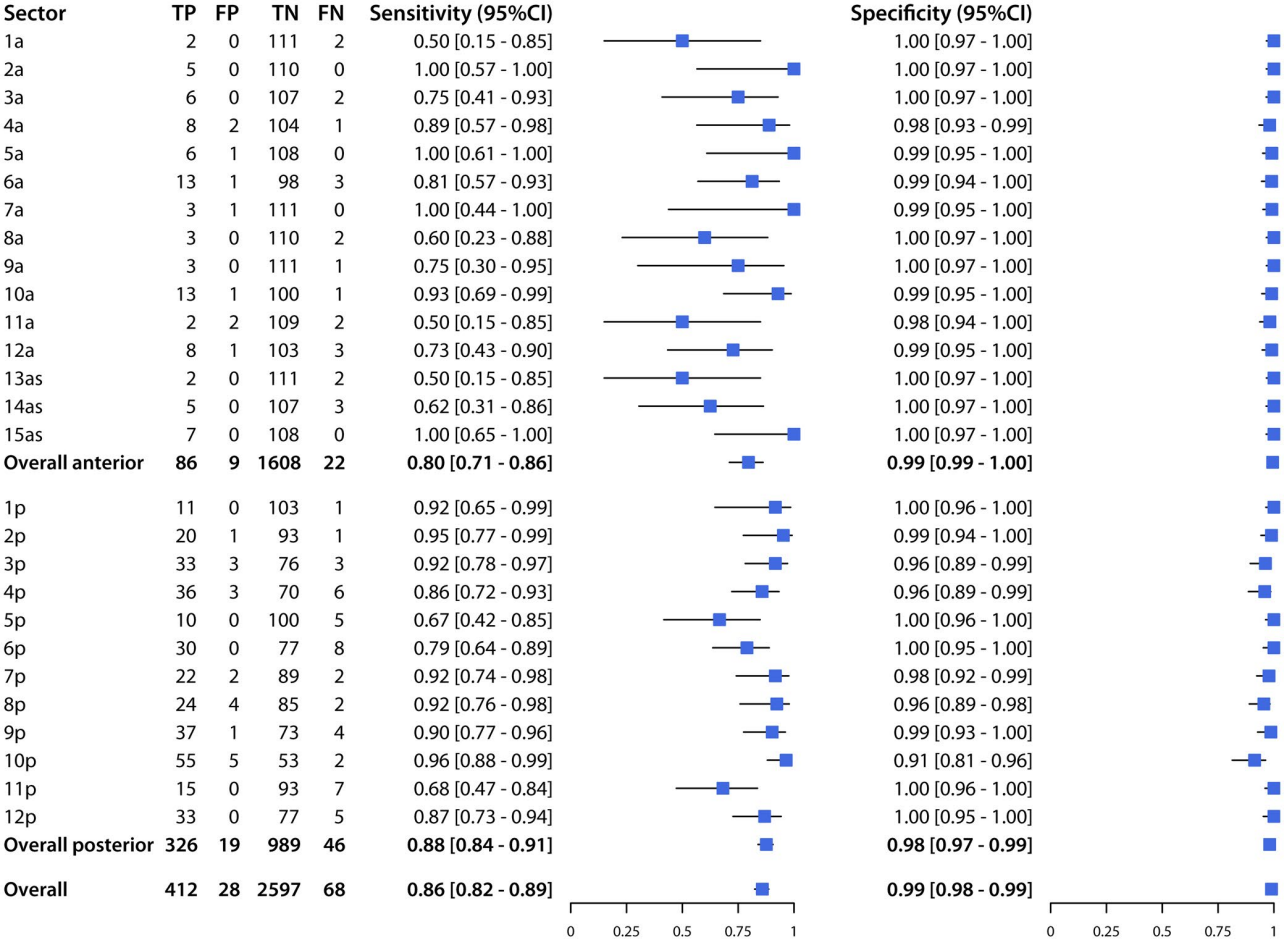
Sensitivity and specificity of mpMRI for detection of PCa (PI‐RADS ≥ 3 and Gleason ≥ 6), for each sector, the anterior prostate, the posterior prostate, and the overall prostate

**FIGURE 3:**
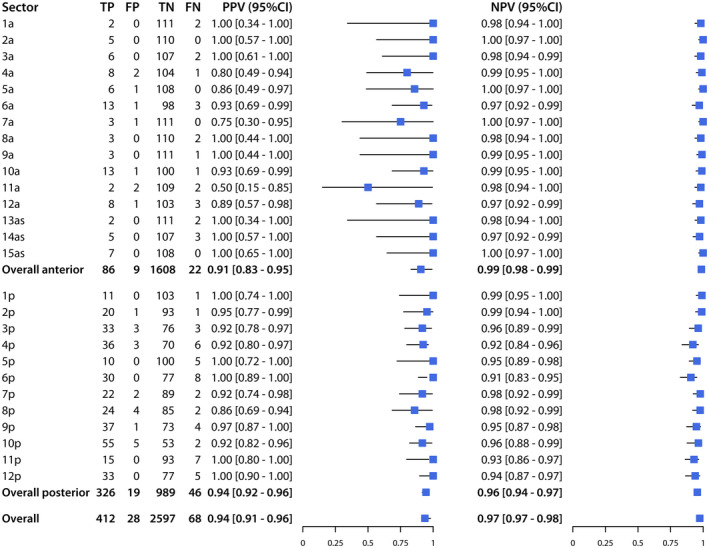
Positive predictive value (PPV) and negative predictive value (NPV) of mpMRI for detection of PCa (PI‐RADS ≥ 3 and Gleason ≥ 6), for each sector, the anterior prostate, the posterior prostate, and the overall prostate

Across the 27 sectors of the prostate, sensitivity ranged from 50% to 100% and specificity from 96% to 100%, while PPV ranged from 50% to 100%, and NPV from 91% to 100% (Figures 2 and 3). The sectors with the lowest sensitivity were 1a (50%; CI, 15%‐80%), 11a (50%; CI, 15%‐85%), and 13as (50%; CI, 15%‐85%), and those with the lowest PPV were 11a (50%; CI,15%‐85%), 7a (75%; CI, 30%‐90%), and 4a (80%; CI, 49%‐94%).

Of the 480 positive sectors according to histopathology, 108 (22%) were in the anterior prostate, while 372 (78%) were in the posterior prostate. For the anterior prostate, mpMRI had a sensitivity of 80% (CI, 71%‐86%), specificity of 99% (CI, 99%‐100%), PPV of 91% (CI, 83%‐95%), and NPV of 99% (CI, 98%‐99%). For the posterior prostate, mpMRI had a sensitivity of 88% (CI, 84%‐91%), specificity of 98% (CI, 97%‐99%), PPV of 94% (CI, 92%‐96%), and NPV of 96% (CI, 94%‐97%).

Overall, mpMRI had a sensitivity of 86% (CI, 82%‐89%), specificity of 99% (CI, 98%‐99%), PPV of 94% (CI, 91%‐96%), and NPV of 97% (CI, 97%‐98%).

### 3.2 Detection of csPCa

For the detection of csPCa (PI‐RADS ≥ 4 and Gleason ≥ 7), histopathology from RP specimens reported 437 positive sectors (14.1%), while mpMRI reported 440 positive sectors (14.7%). There were 386 TPs (12%), 54 FPs (2%), 51 FNs (2%), and 2,614 TNs (84%) (Table 2). The most frequently positive sectors were 10p (n = 54), 4p (n = 40), and 9p (n = 36), and the least frequently positive sectors were 1a, 7a and 11a (n = 3). In the 68 sectors where PCa was not observed on mpMRI (FNs), the Gleason score was never more than 7 (3 + 3 in 17, 3 + 4 in 43, and 4 + 3 in 8).

**FIGURE 4:**
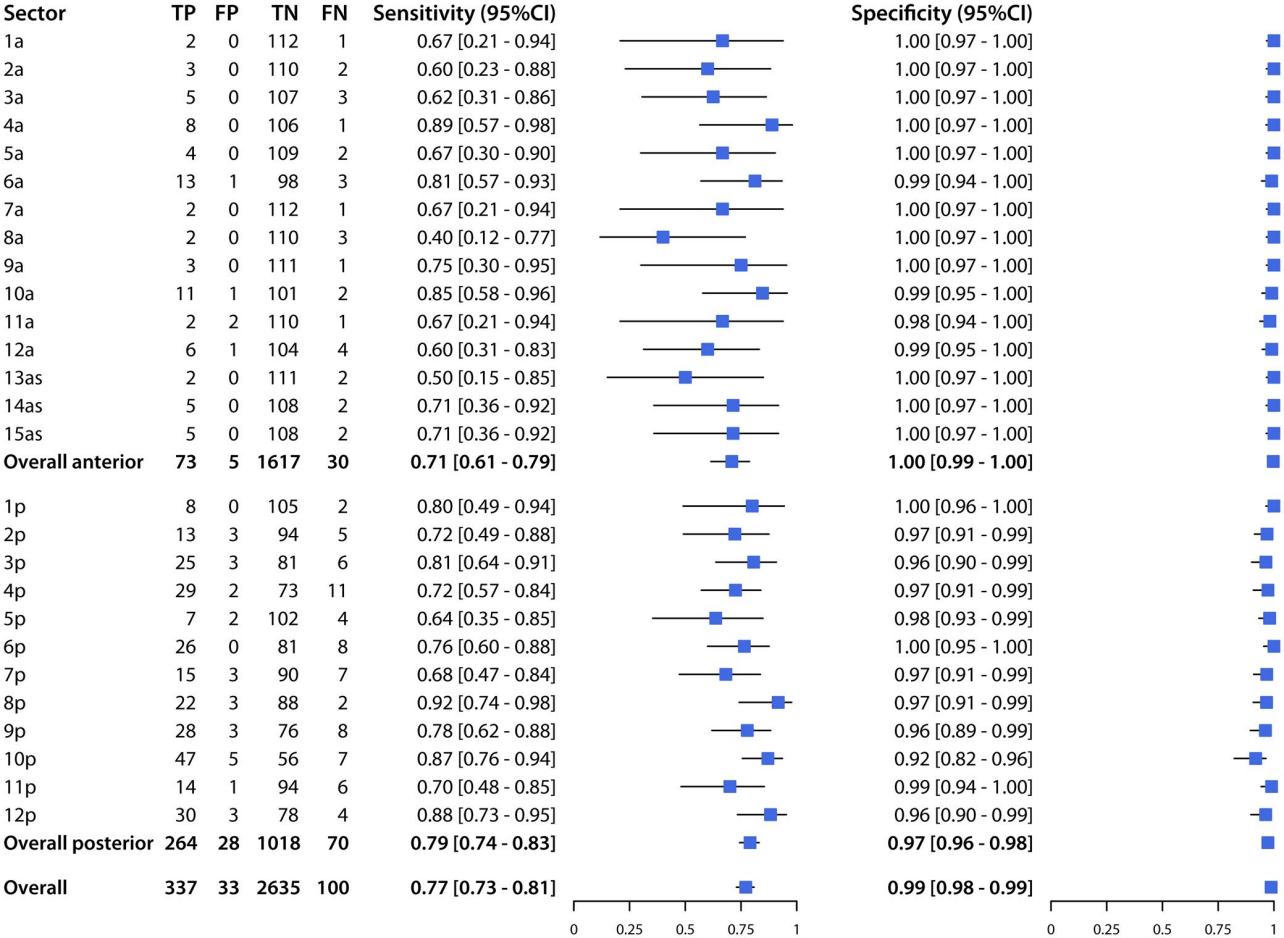
Sensitivity and specificity of mpMRI for detection of csPCa (PI‐RADS ≥ 4 and Gleason ≥ 7), for each sector, the anterior prostate, the posterior prostate, and the overall prostate

**FIGURE 5:**
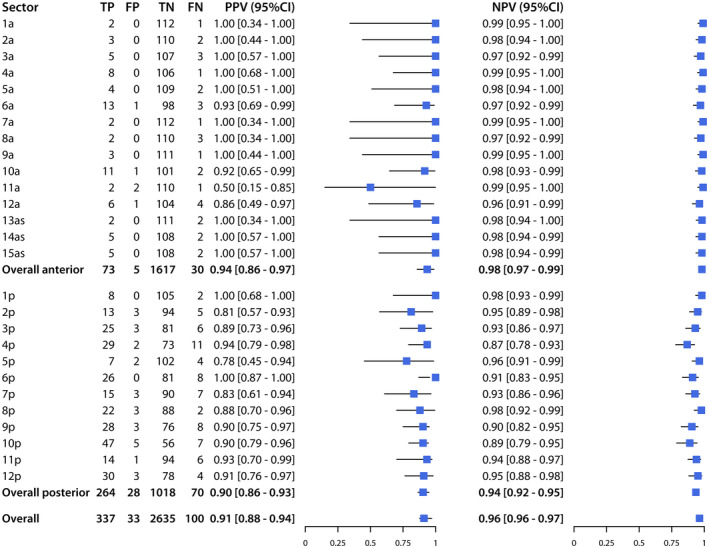
Positive predictive value (PPV) and negative predictive value (NPV) of mpMRI for detection of csPCa (PI‐RADS ≥ 4 and Gleason ≥ 7), for each sector, the anterior prostate, the posterior prostate, and the overall prostate

Across the 27 sectors of the prostate, sensitivity ranged from 40% to 92% and specificity from 92% to 100%, while PPV ranged from 50% to 100%, and NPV from 87% to 99% (Figures 4 and 5). The sectors with the lowest sensitivity were 8a (40%; CI, 12%‐77%) and 13as (50%; CI, 15%‐85%), and those with the lowest PPV were 11a (50%; CI,15%‐85%), 5p (78%; CI, 45%‐94%), and 2p (81%; CI, 57%‐93%).

Of the 437 positive sectors according to histopathology, 103 (24%) were in the anterior prostate, while 334 (76%) were in the posterior prostate. For the anterior prostate, mpMRI had a sensitivity of 71% (CI, 61%‐79%), specificity of 100% (CI, 99%‐100%), PPV of 94% (CI, 86%‐97%), and NPV of 98% (CI, 97%‐99%). For the posterior prostate, mpMRI had a sensitivity of 79% (CI, 74%‐83%), specificity of 97% (CI, 96%‐98%), PPV of 90% (CI, 86%‐93%), and NPV of 94% (CI, 92%‐95%).

Overall, mpMRI had a sensitivity of 77% (CI, 73%‐81%), specificity of 99% (CI, 98%‐99%), PPV of 91% (CI, 88%‐94%), and NPV of 96% (CI, 96%‐97%).

### 3.3 Extraprostatic extension

Histopathology reported EPE in 32 patients (28%), while mpMRI reported 31 (27%). There were 18 TPs (16%), 13 FPs (11%), 14 FNs (12%), and 70 TNs (61%). mpMRI had a sensitivity of 56% (CI, 39%‐72%), and specificity of 84% (CI, 75%‐91%), with a PPV of 58% (CI, 41%‐74%), and NPV of 83% (CI, 74%‐90%).

## 4 DISCUSSION

Using histopathology from RP specimens as the reference standard to diagnose PCa, mpMRI had variable sensitivity (50%‐100%) and PPV (50%‐100%) across the 27 sectors of the prostate, while exhibiting excellent specificity (96%‐100%) and NPV (91%‐100%) in all sectors. The clinical relevance of these findings is that, while mpMRI is not uniformly reliable at ruling out PCa across some sectors of the prostate (with variable probabilities of missing a tumor), mpMRI is uniformly reliable at ruling in PCa in all sectors (with very low probability of indicating a tumor that is not present). The accuracy of mpMRI in detecting and locating prostate tumors depends on the affected region, with particular variability across the anterior sectors, but its high NPV across all sectors suggests that negative findings may not need to be corroborated by randomized biopsy, and that focal therapies can be considered for some cases as a less invasive alternative to RP. The accuracy of mpMRI for detection of csPCa compared to PCa, remains high for specificity and NPV, while it is reduced for sensitivity and PPV.

The results of the present study showed that mpMRI had the highest sensitivity (100%) in sectors 2a, 15as, 7a, and 5a, followed by 10p (96%) and 2p (95%), while it had the lowest sensitivity (50%) in sectors 1a, 11a, and 3as, followed by 8a (60%) and 14as (62%). It is worth noting that the sectors where mpMRI had the highest and lowest sensitivities were also those with the fewest observations of PCa, and hence, the largest confidence intervals. The sensitivity of mpMRI was lower for the anterior prostate (80%; CI, 71%‐86%) than for the posterior prostate (88%; CI, 84%‐91%). Other recent studies reported lower sensitivity for the anterior prostate (62.4% and 78.1%) ^26^, ^27^, which remains the more challenging region to diagnose not only on mpMRI, but also by DRE and/or TRUS. Lawrentschuk et al^28^ identified a subgroup of patients with “prostatic evasive anterior tumor syndrome” (PEATS), whereby anterior predominant tumors remain undiagnosed by DRE and/or biopsy, while Schouten et al^29^ found TRUS biopsy detected only 21% of tumors in the anterior prostate. Therefore, positive mpMRI findings in the anterior prostate should inform clinicians’ decisions to perform targeted biopsies to confirm the presence and location of PCa.

In this study, for the detection of PCa, mpMRI had an overall sensitivity of 86% (CI, 82%‐89%), and an overall specificity of 99% (CI, 98%‐99%) when using 27 sectors and a 3T MRI unit. Wibulpolprasert et al^22^ reported that mpMRI had an overall sensitivity of 28.5% and an overall specificity of 96.3%, when using 39 sectors and a 3T MRI, while Isebaert et al^30^ reported an overall sensitivity of 49.3% and an overall specificity of 86.5% when using 24 sectors and a 1.5T MRI. The differences between findings could be attributed to the use of different sectors maps and MRI systems. In addition, Wibulpolprasert et al. used an endorectal coil for image acquisition, which can distort the prostatic anatomy,^31^ and may have resulted in misregistration when comparing against RP specimens.

The total number of false negative sectors in the present study was 68 of 3105, with the highest number of false negatives occurring in sectors with tumors graded Gleason 3 + 4 (43, 63.2%), followed by Gleason 3 + 3 (17, 25%), and then, Gleason 4 + 3 (8, 11.8%), with no false negatives reported for sectors with tumors graded Gleason ≥4+4. This corresponds with findings reported by Tan et al^32^ and Lee et al,^33^ who concluded that missed lesions on mpMRI were more likely to be lower grade PCa (Gleason 3 + 3 and 3 + 4). Li et al^34^ found a relationship between the genes involved in PCa prognosis (and hence, aggressiveness) and the visibility of such tumors on mpMRI, which could be a cause for under‐detection. Further to this, Troung et al^35^ showed that Gleason 4 patterns with cribeform architecture were often not visualized on mpMRI, unlike Gleason 4 patterns with poorly formed or fused gland architecture. Given that patients with Gleason 3 + 4 cribeform architecture have significantly poorer prognosis^36^ and higher rates of metastatic disease^37^ than patients without cribeform architecture, the use of both targeted and randomized sampling methods for biopsy is recommended to prevent misdiagnosis of lower grade PCa.

For detection of EPE on mpMRI, the present study shows a sensitivity of 56% (CI, 39%‐72%) and a specificity of 84% (CI, 75%‐91%), similar to the findings of a meta‐analysis by de Rooij et al,^38^ which pooled the results of 38 studies and reported a sensitivity of 61% (CI, 54%‐67%), and specificity of 88% (CI, 85%‐91%). With high specificity, mpMRI could be helpful at ruling in EPE, and can therefore advise treatment planning.

This study has a number of limitations, including its retrospective design, as well as the risk of selection bias that is inherent in studying a population of patients who underwent RP, and as such, the sample population does not represent patients that had mpMRI but did not subsequently undergo RP. This may lead to the overestimation of diagnostic accuracy, as the agreement between mpMRI and histopathology results may be “artificially high”.^39^ Furthermore, inter‐rater agreement was not assessed, as mpMRI images were only evaluated by one radiologist, which could result in a lack of quality control, although inter‐rater agreement for PI‐RADS v2 has already been shown to be moderate to substantial.^40^ Finally, there is the potential for mismatch between mpMRI and RP sectors, due to deformation or shrinkage of RP specimens, and the possible misalignment between the axial plane on mpMRI and the sectioning angle used for histopathology specimens. While the limitations of this matching method should be acknowledged, the use of RP as the “gold standard” can be considered a strength of this study, as it provides the highest degree of validation. A new possibility of imaging approach before biopsy, used complimentary to MRI, could be prostate‐specific membrane antigen using positron emission tomography (PSMA PET), which could improve diagnostic sensitivity.

### 4.1 Conclusion

Using histopathology from RP specimens as the reference standard to diagnose PCa on Dickinson's 27‐sector map, mpMRI had variable sensitivity (50%‐100%) and PPV (50%‐100%), while exhibiting excellent specificity (96%‐100%) and NPV (91%‐100%) in all sectors. The accuracy of mpMRI in detecting and locating prostate tumors depends on the affected region, but its high NPV across all sectors suggests that negative findings may not need corroboration by other techniques.

## CONFLICT OF INTEREST

MM, SR, SR, ISQ, GADB, and CHR have no conflicts of interest. LS, GH, and SRP report personal fees from ReSurg SA, during the conduct of the study.
